# Depression and anxiety in children with benign childhood epilepsy with centrotemporal spikes (BCECTS)

**DOI:** 10.1186/s12887-016-0670-2

**Published:** 2016-08-17

**Authors:** Xinjie Liu, Qizheng Han

**Affiliations:** 1 Department of Pediatrics, Qilu Hospital, Shandong University, and Brain Science Research Institute, Shandong University, No. 107 Wen Hua Xi Road, Jinan, People’s Republic of China; 2 Department of Respiratory Medicine, Provincial Hospital Affiliated to Shandong University, No. 4 Duan Xing Xi Road, Jinan, People’s Republic of China

**Keywords:** Benign childhood epilepsy with centrotemporal spikes (BCECTS), Depression, Anxiety, Childhood

## Abstract

**Background:**

Elevated rates of affective disturbance in children with benign childhood epilepsy with centrotemporal spikes (BCECTS) have been reported. However, it remains unclear how anxiety and depression are related to epilepsy, and it is unknown whether these mood disorders are influenced by the use of antiepileptic drugs. In the present report, we performed a prospective study designed to evaluate affective disorders (anxiety and depression) without the bias of antiepileptic drug treatment in 89 children with BCECTS, based on self-reporting. Furthermore, we sought to determine whether clinical factors, such as age, disease course, seizure frequency, and spike wave index (SWI), were related to the psychological profiles.

**Methods:**

Patients with BCECTS (*n* = 89) and healthy matched controls (*n* = 75) were included in this study. The Depression Self-Rating Scale for Children (DSRSC) and the Screen for Child Anxiety-Related Emotional Disorders (SCARED) were completed by the children.

**Results:**

None of the children met criteria for clinically significant anxiety or depression. However, the children with BCECTS had significantly higher depression and anxiety scores compared with children in the control group. We found no significant differences in depression or anxiety between the left, right, and bilateral lobe groups. The DSRSC scores were similar between the children with partial seizures and those with secondarily generalized seizures. Similarly, there were no significant differences in the SCARED scores between these two groups. However, the DSRSC and SCARED scores were positively correlated with age, seizure frequency, SWI, and disease course.

**Conclusions:**

The children with BCECTS had an increased likelihood of depression and anxiety, and these higher rates were unrelated to seizure type or epileptic focus, but were positively correlated with age, seizure frequency, SWI, and disease course.

## Background

Epilepsy is a common childhood neurological disorder often associated with impaired behavioral, emotional and/or cognitive functioning. A growing body of literature has demonstrated that children with epilepsy face numerous challenges, including a high burden of psychiatric and behavioral comorbidities that often go untreated or even unrecognized [1–4]. Benign childhood epilepsy with centrotemporal spikes (BCECTS, also known as benign Rolandic epilepsy) is the most frequent form of epilepsy in childhood [5], and about 15 % of all children with epilepsy have BCECTS [6]. Because of the high frequency of BCECTS among all cases of pediatric epilepsy, special attention has been given to the rate of neuropsychiatric comorbidities in this condition. Some studies have found below average school performance and various behavioral comorbidities, including attention-deficit hyperactivity disorder, in children with BCECTS [7–10]. Elevated rates of affective disturbance have also been reported [11–13]. However, it is unclear how the anxiety and depression are related to epilepsy, and it is unknown whether antiepileptic drugs contribute to these behavioral disorders. Most published studies include a diverse mix of patients treated with different medications as well as untreated patients. Medication side effects, therefore, may have affected emotional functioning. Moreover, relatively small sample sizes may possibly have biased the results. In addition, most previous studies relied on parent reports rather than direct neuropsychological or psychiatric evaluation of the children. The present prospective study was therefore designed to evaluate affective disorders (anxiety and depression) without the bias of antiepileptic drug treatment in 89 BCECTS children, based on reports from the children themselves. We also assessed whether clinical factors, such as age, disease course, seizure frequency, and spike wave index (SWI), were related to the psychological profiles.

## Methods

### Participants

This study protocol was submitted to and approved by the Institutional Research Ethics Committee of Qilu Hospital, Shandong University, and approval from the parents of each of the participants was acquired. All parents and children gave verbal informed consent. This study also adhered to STROBE guidelines.

The study included 89 BCECTS children aged 8–14 years, with IQ scores of 75 and higher, and 76 non-BCECTS children. All BCECTS children were recruited from the pediatric epilepsy outpatient department, and the primary study inclusion criteria for each subject were a diagnosis of BCECTS according to the ILAE criteria (partial, hemifacial motor seizures frequently having associated somatosensory symptoms … often related to sleep. Onset occurs between the ages of 3 and 13 years) [14] and at least three seizures within the year prior to participation in the study. All BCECTS children exhibited normal background electroencephalograms (EEGs), and their SWIs during non-rapid eye movement were less than 50 %. The recruited patients had never received antiepileptic drug treatment prior to participation in the study. We excluded patients with concomitant neurological or psychiatric disorders, vision or hearing deficits, or abnormal magnetic resonance imaging findings.

We recruited 76 healthy age-, sex-, education-, and socioeconomic status-matched children for the control group. Healthy children recruited from local elementary and middle schools. Children and their parents provided verbal consent.

### EEG analysis

Routine EEGs, including recordings during sleep, were performed one or two times on all patients, as described previously by our group [10]. EEGs were obtained in our laboratory using the standard international 10–20 system of EEG electrode positioning (Nihon Kohden, Tokyo, Japan). To enable the detection of a possible association between spike density and mood disorders, we calculated a spike-wave index (SWI) as follows: the number of seconds in which one or more spike waves was present during the first 30 min of the non-rapid eye movement stages of the first and last sleep cycles divided by 3 600 and then multiplied by 100. The results are expressed as percentages [10, 13].

### Depression Self-Rating Scale for Children (DSRSC)

The DSRSC [15] was used to measure the depression status of the children with epilepsy. The DSRSC is a self-report questionnaire that contains 18 items. Each of the 18 items was scored on a 3-point scale (0 = never, 1 = sometimes, 2 = often). The total scores, ranging from 0 to 36, are reported. Low scores reflect the absence of depression, and scores > 15 are considered to indicate a depressive state [16]. In China, the psychometric properties of the DSRSC have been found to be satisfactory, with good reliability and validity (the test-retest reliability ranges from 0.53 to 0.65, and the spilt reliability and Cronbach’s α have been reported to be 0.72 and 0.73, respectively, for ages 8–16 years) [16].

### The Screen for Child Anxiety-Related Emotional Disorders (SCARED)

The SCARED [17] was created by Birmaher to assess anxiety disorders in children, and is a tool that aids psychologists in diagnoses, scientific research, and epidemiological investigations. The Chinese version of this self-report tool consists of 41 items in the following five subscales: somatic/panic disorder, generalized anxiety disorder, separation anxiety disorder, social phobia, and school phobia. There were three response options (0, 1, and 2) for each item, and the total for each subscale was 82. Higher scores indicate higher anxiety severity. The cut-off score for the SCARED is 23. Acceptable reliability and validity have been established for this scale in China (the test-retest reliability ranges from 0.567 to 0.608, the spilt reliability is 0.88, and Cronbach’s α ranges from 0.43 to 0.89, for ages 8–16 years) [18].

### Statistical analyses

SPSS 13.0 was used for all statistical analyses (SPSS Inc., Chicago, IL, USA). All of the results are presented as the mean ± standard deviation (SD). Differences in the scores between the epilepsy group and the control group were analyzed with the Mann-Whitney rank sum test. Nonparametric Kruskal-Wallis tests followed by Bonferroni-corrected Mann-Whitney U tests were performed to compare the DSRSC and SCARED scores between multiple groups. Spearman correlation tests were performed to determine whether there were any correlations between the ages of the children with epilepsy, disease course, seizure frequency, SWI, or affective disorders. A value of *P* < 0.05 was considered statistically significant.

## Results

### Subjects

Eighty-nine children (54 boys, 35 girls) with BCECTS aged 8–14 years participated in this study. There were no significant differences in education or socioeconomic status between the controls and the BCECTS children. The clinical and electroclinical characteristics of the children with BCECTS are summarized in Table 1.

**Table 1:** General clinical data of the 89 children with BCECTS

Characteristics	Number
Sex	
Male	54 (60.7 %)
Female	35 (39.3 %)
Age (8 ~ 14 years)	
≤ 10	38 (42.7 %)
11 ~ 12	30 (33.7 %)
≥ 13	21 (23.6 %)
Duration of illness (years) $\left(\overline{x}\pm s\right)$	2.8 ± 2.2
Seizure frequency (per year)	
< 5	26 (29.2 %)
5–10	45 (50.6 %)
> 10	18 (20.2 %)
Seizure types	
Partial	52 (58.4 %)
Secondary generalized	37 (41.6 %)
Epileptic focus	
Left	35 (39.3 %)
Right	38 (42.7 %)
Bilateral	16 (18.0 %)
SWI (6–43 %)	
≤ 25 %	43 (48.3 %)
> 25 %	46 (51.7 %)

### Comparison of the DSRSC and SCARED scores between the BCECTS and control groups

We defined depression as a DSRSC score greater than 15, and anxiety as a SCARED score greater than 23. Although none of the 89 children with BCECTS met the criteria for diagnoses of anxiety or depression, they had significantly higher depression and anxiety scores than the children in the control group. DSRSC and SCARED scores were similar between male and female individuals. The DSRSC and SCARED scores for the controls and epileptic children are presented in Tables 2 and 3.

**Table 2:** DSRSC scores of the children in the control and BCECTS groups

DSRSC Scales	Control Group (*n* = 76)	BCECTS Group (*n* = 89)
1. I look forward to things as much as I used to	1.12 ± 0.79	1.20 ± 0.73
2. I sleep very well	1.48 ± 0.65	1.43 ± 0.72
3. I feel like crying	0.35 ± 0.55	0.96 ± 0.68^**^
4. I like to go out to play	1.12 ± 0.72	1.26 ± 0.74
5. I feel like running away	0.59 ± 0.66	0.56 ± 0.43
6. I get tummy aches	0.78 ± 0.65	0.82 ± 0.58
7. I have lots of energy	0.89 ± 0.45	1.18 ± 0.76^**^
8. I enjoy my food	0.96 ± 0.63	1.32 ± 0.75^**^
9. I can stick up for myself	0.96 ± 0.58	1.35 ± 0.70^**^
10. I think life isn't worth living	0.29 ± 0.49	0.85 ± 0.46^**^
11. I am good at things I do	0.55 ± 0.51	0.82 ± 0.53^**^
12. I enjoy the things I do as much as 1 used to	0.73 ± 0.50	0.88 ± 0.75
13. I like talking with my family	0.69 ± 0.65	0.97 ± 0.72^*^
14. I have horrible dreams	0.52 ± 0.48	0.76 ± 0.42
15. I feel very lonely	0.59 ± 0.38	0.87 ± 0.40^**^
16. I am easily cheered up	1.04 ± 0.57	1.12 ± 0.67
17. I feel so sad I can hardly stand it	0.25 ± 0.46	0.83 ± 0.56^**^
18. I feel very bored	0.45 ± 0.68	0.95 ± 0.70^**^
Total DSRSC scores	13.0 ± 10.40	19.43 ± 13.65^**^

* *P* < 0.05, ** *P* < 0.01

**Table 3:** SCARED scores of the children in the control and BCECTS groups

SCARED Scales	Control Group (*n* = 76)	BCECTS Group (*n* = 89)
Somatic/panic	2.97 ± 2.56	4.32 ± 3.73^*^
Generalized anxiety	3.02 ± 2.65	6.68 ± 3.89^*^
Separation anxiety	3.56 ± 2.55	5.35 ± 2.34^*^
Social phobia	3.62 ± 2.76	6.05 ± 2.74^*^
School phobia	0.89 ± 0.96	2.05 ± 1.43^*^
Total DSRSC scores	14.40 ± 10.40	25.40 ± 13.65^*^

The asterisks indicate statistically significant differences between the controls and BCECTS patients (*P* < 0.01)

### Comparison of the DSRSC and SCARED scores among children with different spike locations

To examine the possible effect of spike location on mood, we examined the DSRSC and SCARED scores according to affected lobe. We found no significant differences in depression or anxiety among the left, right, and bilateral lobe groups (Table 4).

**Table 4:** DSRSC and SCARED scores of the BCECTS children according to the spike location

Group	Number	DSRSC scores	SCARED scores
Left lobe	35	19.47 ± 7.26	25.96 ± 10.53
Right lobe	38	19.55 ± 7.44	24.64 ± 13.35
Bilateral lobes	16	18.56 ± 8.92	24.25 ± 12.90
*F* value		2.89	3.0
*P*		> 0.05	> 0.05

### Comparison of the DSRSC and SCARED scores among children with different seizure types

The influence of seizure type on the DSRSC and SCARED scores was investigated. The DSRSC scores were similar between the children with partial seizures and those with secondarily generalized seizures. Similarly, there were no significant differences in the SCARED scores between these two groups (Table 5).

**Table 5:** DSRSC and SCARED scores of the BCECTS children according to seizure type

Groups	Number	DSRSC Scores	SCARED Scores
Partial seizure	52	19.47 ± 8.26	25.96 ± 12.53
Secondarily generalized seizure	37	19.55 ± 7.44	25.64 ± 13.35
*t* value		0.05	0.11
*P*		> 0.05	> 0.05

### The relationship between clinical factors and DSRSC and SCARED scores

To investigate possible correlations between clinical factors (i.e., age, seizure frequency, SWI, and disease course) and affective disorders, analyses within the BCECTS group were performed using Spearman’s rank correlation. The DSRSC scores were positively correlated with age, seizure frequency, SWI, and disease course. Similarly, positive correlations were found between the SCARED scores and each of the four different clinical factors (Table 6).

**Table 6:** The Spearman correlation between the clinical factors and affective disorders (*r* value)

Tests	Age	Seizure frequency	SWI	Disease course
DSRSC	0.35*	0.51*	0.37*	0.43*
SCARED	0.42*	0.76*	0.29*	0.54*

* P < 0.05

## Discussion

The major advantage of the present study is the exclusion of patients receiving anti-epileptic treatment. Drug treatment can alter EEG activity. There is also evidence that some antiepileptic drugs may directly induce cognitive dysfunction, thereby masking potential relationships between the disease and cognitive impairments. In this study, we examined the depression and anxiety scores of children with BCECTS and found that BCECTS was associated with increased risks of depression and anxiety [19], although none of the children with BCECTS met the criteria for diagnoses of anxiety or depression. Additionally, we examined the correlation between the location of the spikes and mood disorders, and found no significant difference in depression or anxiety between the left, right, and bilateral lobe groups, consistent with previous studies [20, 21]. Similarly, we did not identify seizure type as a predictor of emotional problems.

At present, the relationships between comorbidities and seizure type are unclear. Caplan et al. [22] observed that depression is more frequent in children with complex partial seizures, and that anxiety disorders are more frequent in children with childhood absence epilepsy. However, other studies [23, 24] found no significant difference between scales scores and seizure type in children with epilepsy (i.e., partial generalized and undetermined). This discrepancy in findings is likely owing to selection bias (i.e., different inclusion criteria, particularly the inclusion of more or fewer atypical cases and wide age ranges), limited sample sizes, and differences in assessment tools [13].

We also investigated the differential effects of age, disease course, and seizure frequency on psychological profiles. These three factors were found to be correlated with mental health problems. This finding does not fully agree with the findings of previous studies. Although Connolly et al. found that the age of epilepsy onset, duration of epilepsy, and seizure severity were not correlated with the quality of life of children with benign Rolandic epilepsy, children who experienced fewer seizures in the preceding 6 months exhibited lower scores in 3 of the 16 scales (i.e., depression, stigma, and general health) of the Quality of Life in Childhood Epilepsy Questionnaire, compared with children who had not experienced seizures [12]. Samaitiene et al. also reported significantly higher Child Behavior Checklist scores (compared with a control group of patients), but for only the anxiety/depression items, in patients with Rolandic epilepsy who had experienced seizures within the preceding 6 months. In contrast, the Child Behavior Checklist scores of patients with Rolandic epilepsy who had not had seizures in the preceding 6 months did not differ significantly from those of the control group. These findings suggest that active epilepsy affects behavior [25].

The potential influence of frequency and lateralization of centrotemporal spikes (CTS) on cognition and behavior is not well understood [6]. Another aim of our study was to determine whether there were any relationships between SWI and affective problems. We found a significant effect of SWI on depression and anxiety. This suggests that increased epileptiform activity in children with BCECTS may predict higher rates of mood and behavioral problems [16]. However, it remains unknown how epileptiform spike discharges are linked to mood. Similar anatomic hippocampal changes are observed in depression and epilepsy, which suggests a relation between the two disorders and a common functional role of the temporal lobe [26]. However, the central and mid-temporal loci of the characteristic discharges in BCECTS can disrupt neural circuits involving the temporal lobe and cause greater mood dysregulation [27]. Recent animal studies have also revealed that epileptic discharges can evoke depressive-like symptoms [28].

Sleeping difficulties are a common comorbidity in epilepsy, and sleep impairment can also influence behavior and mood. Samaitiene et al. found a positive relationship between sleep and behavioral problems. They found significantly greater behavioral problems in patients with Rolandic epilepsy with sleep problems than in patients with Rolandic epilepsy without sleep problems [25]. Furthermore, children with BCECTS experience unrefreshing sleep, which may also contribute to higher depression/anxiety scores.

This study has several limitations. One limitation is the collection of data from a grade III, class A hospital. Therefore, these samples may not represent the entire Chinese BCECTS population. In addition, the parents who visited the hospital were usually in a relatively better economic state and more concerned about this disorder. Finally, no longitudinal investigation was performed. Despite these limitations, there are still a number of major clinical implications of our study. Our findings demonstrate that children with BCECTS, especially older children, have a longer duration of epilepsy, have more frequent seizures, and often exhibit affective disturbance requiring intervention.

## Conclusion

The present study demonstrates that children with BCECTS exhibit an increased likelihood of depression and anxiety, and these higher rates are unrelated to seizure type or epileptic focus. Moreover, these affective disorders were found to be positively correlated with age, seizure frequency, SWI, and disease course. Therefore, it is important that children with BCECTS undergo regular psychiatric assessment and that therapeutic interventions are initiated as early as possible.

## Abbreviations

BCECTS, benign childhood epilepsy with centrotemporal spikes; DSRSC, depression self-rating scale for children; SCARED, screen for child anxiety-related emotional disorders; SWI, spike wave index
